# Obstinate Hæmorrhage from the Extraction of Teeth

**Published:** 1848-01

**Authors:** 


*Obstinate Hæmorrhage from the Extraction of Teeth*—A gentleman, about twenty-one or twenty-two years of age, called upon us a few weeks since, who, two days before, had had the first left superior molaris extracted by a dentist at a country village, some twenty or twenty-five miles from Baltimore. The hæmorrhage which immediately followed the operation, was not great, and in a few minutes nearly subsided, but in a few hours it become so profuse that he was induced to call on the dentist again, who applied from time to time, for more than twelve hours, the various means usually employed for arresting hæmorrhages of this description, but without success. The bleeding continued until he become so much reduced, that serious apprehensions were entertained with regard to the consequences likely to result from it.

Under the above circumstances he came to the city. He arrived about eight o'clock in the evening, and applied to us immediately for relief. Upon examining his mouth, after freeing the alveolus from the partially coagulated blood, we found that the bleeding was principally from the socket and inner margin of the gum. We now filled the socket with pledgets of raw cotton, saturated in tinct. nut galls, packing them as closely as possible; after which, we applied a thick compress. This last was held in place by closing the teeth of the lower jaw firmly against it. A strong bandage was now applied under the chin and over the top of the head in such a manner as to prevent the lower jaw from being depressed.

The hæmorrhage immediately ceased, but about five o'clock the next morning, the bandage becoming partially loosened, the bleeding commenced again, and continued for more than two hours before I was sent for. The blood now seemed to come from the inner edge of the gum where it had been separated from the alveolar border. Compresses were again applied together with the most powerful styptics, but to no purpose.

The application of the actual cautery, now seemed to be the last resort, and not being provided with a steel or iron instrument of a proper shape, the patient being at a boarding house in the immediate vicinity of Professor Smith's, I requested this eminent surgeon to see him with me, and to come provided with one that might be employed for that purpose. This he did, and concurring in the opinion that the actual cautery should be used, it was immediately applied and at once put a stop to the hæmorrhage.

The foregoing is the only case which has ever fell under our own immediate observation in which hæmorrhage from the extraction of a tooth could not be promptly arrested by the application of a suitable compress introduced into the socket. We have had a number of cases, in which, from a constitutional hæmorrhagic diathesis, the bleeding was so profuse and continued so long as to excite a great deal of alarm, but never before failed to arrest it by plugging the socket with sponge or raw cotton saturated with some strong liquid astringent. The immediate replacement of the tooth into the alveolus, is recommended by some writers. The mouths of the bleeding vessels may, unquestionably, be more thoroughly compressed by this means, but a plug of raw cotton or sponge, will, in most cases, be found equally efficient, and as it is productive of less mechanical injury, it should, as a general rule be preferred.

